# Generalized Effective Medium Theory for Particulate Nanocomposite Materials

**DOI:** 10.3390/ma9080694

**Published:** 2016-08-13

**Authors:** Muhammad Usama Siddiqui, Abul Fazal M. Arif

**Affiliations:** Mechanical Engineering Department, King Fahd University of Petroleum & Minerals, Dhahran 31261, Saudi Arabia; musiddiqui@gmail.com

**Keywords:** nanocomposite, effective medium theory, thermal conductivity estimation, multiple inclusions, non-uniform dispersion

## Abstract

The thermal conductivity of particulate nanocomposites is strongly dependent on the size, shape, orientation and dispersion uniformity of the inclusions. To correctly estimate the effective thermal conductivity of the nanocomposite, all these factors should be included in the prediction model. In this paper, the formulation of a generalized effective medium theory for the determination of the effective thermal conductivity of particulate nanocomposites with multiple inclusions is presented. The formulated methodology takes into account all the factors mentioned above and can be used to model nanocomposites with multiple inclusions that are randomly oriented or aligned in a particular direction. The effect of inclusion dispersion non-uniformity is modeled using a two-scale approach. The applications of the formulated effective medium theory are demonstrated using previously published experimental and numerical results for several particulate nanocomposites.

## 1. Introduction

Typically, the process of heat conduction is treated using the classical Fourier’s law. Although Fourier’s law is widely applied, its application to systems with characteristic lengths comparable to or lower than the mean-free-path of the energy carriers (phonons or electrons) leads to large errors in any or all variables in the system such as the thermal conductivity, temperature and the temperature gradient [1]. Examples of such systems include nanoparticles, nanowires and thin films. The reason for the inapplicability of Fourier’s law for nanostructures is that during heat conduction in such systems, equilibrium conditions are not achieved and therefore, a local temperature gradient is not established. Without a temperature gradient, Fourier’s law is not applicable and thermal conductivity, which relates heat flux to the temperature gradient, has no meaning. However, if one is interested in heat conduction in a domain with a characteristic length much greater than the characteristic length of the nanostructures, Fourier’s law may still be applied using an effective value of thermal conductivity. An example of such a case is a nanocomposite where the domain under consideration is considerably larger than the characteristic length of the nanoparticles.

The problem of estimation of the effective thermal conductivity of composite materials has been widely studied. Early works in the area were done by Maxwell [2] and Lord Rayleigh [3] who studied the thermal conductivities of composites with low concentrations of inclusions. Their works were later extended by Hasselman and Johnson [4] and Benveniste [5] who studied the effects of thermal boundary conductance on the effective thermal conductivity of the composite. Bruggeman [6] derived a model for the effective thermal conductivity of a composite when the inclusion concentration was high. His model was later extended by Every and coworkers [7] to include the effect of thermal boundary conductance. Modifications to the model by Every and coworkers to include the effect of particle shape have also been presented [8]. An effective medium theory (EMT) for the estimation of thermal conductivity of composites with dilute concentrations of inclusions of different shapes was presented by Nan and coworkers [9]. The major drawbacks of the effective medium theory approach discussed above include the limitation of using inclusions of regular shapes, inability to handle non-uniformly dispersed and percolating inclusions and inability to deal with nanocomposites [1]. To overcome the inability to handle percolating networks of inclusions, Prasher and coworkers [10,11] and Evans and coworkers [12] presented a three-level homogenization methodology capable of handling clustering of inclusions.

The application of the models mentioned above to nanocomposites can lead to significant errors in the predicted effective thermal conductivity. Many times, the addition of nanoparticles in a matrix can result in an effective thermal conductivity which is significantly lower than the thermal conductivities of both the matrix and the inclusion [13,14]. The use of conventional modeling approaches for such composites results in an overestimation of the effective thermal conductivity even when using very high interface thermal resistance [15,16]. Wu and coworkers [15] used Nan and coworkers’ model [17] to estimate the thermal conductivity of aluminum matrix composites with carbon nanotube (CNT) inclusions and found that the model over-predicted the results even when the thermal interface resistance was set to infinity. Ahmad and coworkers [16] carried out a similar study for alumina–CNT composite and arrived at the same conclusion.

To reduce the errors in effective medium theory predictions for nanocomposites, a modified effective medium theory was presented by Minnich and Chen [18] for spherical inclusions and was extended by Ordonez-Miranda and coworkers [19] for spheroidal inclusions. In their approach, modified thermal conductivities of the matrix and inclusions are first calculated and then used in the effective medium theory. Minnich and Chen used the modified values of matrix and inclusion thermal conductivities in Nan and coworkers’ EMT for spherical inclusions and found good agreement between the effective thermal conductivities predicted by the modified EMT and Monte Carlo simulations. The shortcomings of the modified effective medium theory approach of Minnich and Chen and Ordonez-Miranda and coworkers include the inability to handle multiple inclusions, randomly oriented inclusions, and non-uniformly distributed inclusions. Other approaches used for the estimation of effective thermal conductivity of nanocomposites include the Monte Carlo simulation method [14,20,21], molecular dynamics [22] and the Boltzmann transport equation [23,24,25,26,27].

**Figure 1:**
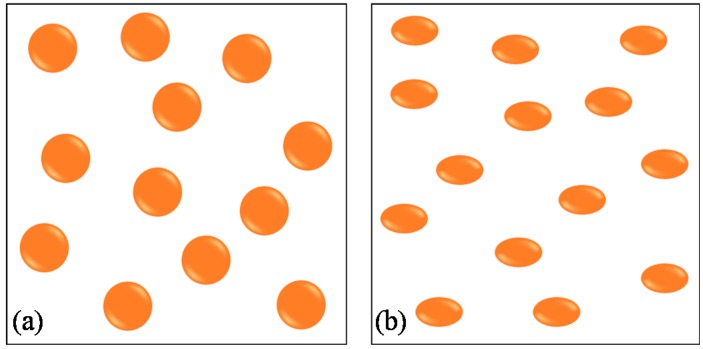

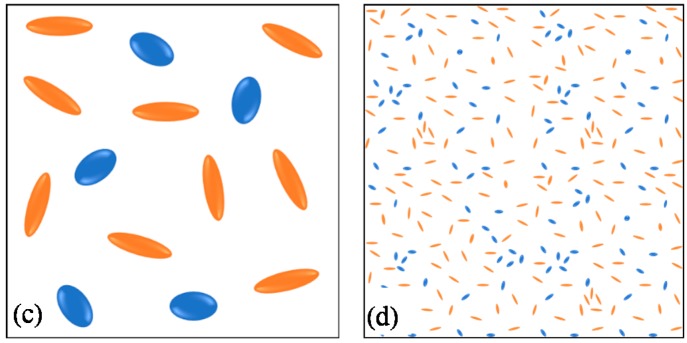
Inclusion geometries and orientation for: (**a**) Minnich and Chen [18]; (**b**) Ordonez-Miranda and coworkers [19]; and (**c**) generalized EMT—current work; (**d**) A schematic representation non-uniformly distributed inclusions—current work (inclusion sizes are not to scale).

The current work shows the formulation of a generalized effective medium theory which can be used to determine the effective thermal conductivity of particulate nanocomposites. The proposed effective medium theory overcomes several shortcomings in the effective medium theory approaches reported in the literature. These include capabilities to include the effect of multiple nanometer-sized inclusions, the effect of oriented (randomly or at any angle relative to heat flow direction) spheroidal, cylindrical or platelet inclusions and the effect of non-uniformly dispersed inclusions. Figure 1 graphically shows the capability of the formulated generalized effective medium theory in comparison to those proposed by Minnich and Chen [18] and Ordonez-Miranda and coworkers [19]. The formulation of the generalized effective medium theory is presented in Section 2 while Section 3 shows the application of the formulated EMT.

## 2. Framework of the Generalized Effective Medium Theory

The formulation of the generalized effective medium theory is presented in this section. First, the effective medium theory formulation for multiple inclusions is presented in Section 2.1. Second, the effect of multiple nanometer-sized inclusions on the matrix and inclusion thermal conductivities is derived in Section 2.2. Lastly, a two-scale approach to handle non-uniformly dispersed inclusions is presented in Section 2.3.

### 2.1. Effective Medium Theory for Composites with Multiple Inclusions

To derive the effective medium theory for composites with multiple inclusions, a two-phase composite was first considered. The thermal conductivity of the composite was assumed to vary from point to point according to the function $K\left(r\right)=K^{0}+\sum_{n}\delta K_{n}\left(r\right)$ where $K^{0}$ is the constant part of thermal conductivity function and $\delta K_{n}\left(r\right)$ is the fluctuation in thermal conductivity due to inclusion particle *n*. The effective thermal conductivity of the composite was then defined by Equation (1) [28].

(1) $$K_{eff}=K^{0}+\langle T\rangle {\left(I+\langle GT\rangle \right)}^{-1}$$

where I is the unit tensor, **G** is the Green’s tensor [29] and 〈 〉 denotes volumetric average.

The tensor **T**, defined by Equation (2) is the transition matrix and it describes the effect of individual inclusion particles on the effective thermal conductivity of the composite.

(2) $$T=\sum_{n}T_{n}+\sum_{n,m\neq n}T_{n}GT_{m}+\ldots$$

If the inclusion volume fraction is low, **K**^0^ can be taken as the matrix thermal conductivity **K***_mat_*, the fluctuating part becomes **K***_inc_* − **K***_mat_* and **T** can be approximated as,

(3) $$T≅\sum_{n}T_{n}=\sum_{n}\delta K_{n}{\left(I-G\delta K_{n}\right)}^{-1}$$

Using this approach, Nan and coworkers [9] formulated the effective medium theory of composites with spheroidal inclusions of one type. In the current work, their approach was used to extend the methodology for multiple inclusions. First, Equation (3) was extended to Equation (4) for multiple types of inclusions. The effective thermal conductivity of the composite was then calculated using Equations (5)–(13) which are valid for spheroidal inclusions of multiple types.

(4) $$T≅\sum_{i}\sum_{n}T_{inc.i,n}=\sum_{i}\sum_{n}\delta K_{inc.i,n}{\left(I-G\delta K_{inc.i,n}\right)}^{-1}$$

(5) $$\begin{array}{l} K_{eff,11}=K_{eff,22}=K_{mat}\frac{2+\sum_{i=1}^{N}\varphi_{i}\left[\beta_{11}^{i}\left(1-L_{11}^{i}\right)\left(1+{\langle \cos^{2}\theta \rangle }^{i}\right)+\beta_{33}^{i}\left(1-L_{33}^{i}\right)\left(1-{\langle \cos^{2}\theta \rangle }^{i}\right)\right]}{2-\sum_{i=1}^{N}\varphi_{i}\left[\beta_{11}^{i}L_{11}^{i}\left(1+{\langle \cos^{2}\theta \rangle }^{i}\right)+\beta_{33}^{i}L_{33}^{i}{\left(1-\langle \cos^{2}\theta \rangle \right)}^{i}\right]}\\ K_{eff,33}=K_{mat}\frac{1+\sum_{i=1}^{N}\varphi_{i}\left[\beta_{11}^{i}\left(1-L_{11}^{i}\right)\left(1-{\langle \cos^{2}\theta \rangle }^{i}\right)+\beta_{33}^{i}\left(1-L_{33}^{i}\right)\left({\langle \cos^{2}\theta \rangle }^{i}\right)\right]}{1-\sum_{i=1}^{N}\varphi_{i}\left[\beta_{11}^{i}L_{11}^{i}\left(1-{\langle \cos^{2}\theta \rangle }^{i}\right)+\beta_{33}^{i}L_{33}^{i}{\langle \cos^{2}\theta \rangle }^{i}\right]} \end{array}$$

(6) $$\begin{array}{l} K_{c,11}^{i}=\left\{ \begin{array}{l} K_{inc.i}/\left(1+\gamma_{11}^{i}L_{33}^{i}K_{inc.i}/K_{m}\right),\;\text{for platelet inclusions}\\ K_{inc.i}/\left(1+\gamma_{11}^{i}L_{11}^{i}K_{inc.i}/K_{m}\right),\;\text{for other shapes} \end{array}\right.\\ K_{c,33}^{i}=\left\{ \begin{array}{l} K_{inc.i}/\left(1+\gamma_{33}^{i}L_{11}^{i}K_{inc.i}/K_{m}\right),\;\text{for cylindrical inclusions}\\ K_{inc.i}/\left(1+\gamma_{33}^{i}L_{33}^{i}K_{inc.i}/K_{m}\right),\;\text{for other shapes} \end{array}\right. \end{array}$$

(7) $$L_{11}^{i}=L_{22}^{i}=\left\{ \begin{array}{l} \frac{p^{i^{2}}}{2\left(p^{i^{2}}-1\right)}-\frac{p^{i}}{2{\left(p^{i^{2}}-1\right)}^{3/2}}\cosh^{-1}p^{i},\;\text{for}\;p^{i}\geq 1\\ \frac{p^{i^{2}}}{2\left(p^{i^{2}}-1\right)}+\frac{p^{i}}{2{\left(1-p^{i^{2}}\right)}^{3/2}}\cos^{-1}p^{i},\;\text{for}\;p^{i}<1 \end{array}\right.$$

(8) $$\beta_{kk}^{i}=\frac{K_{c,kk}^{i}-K_{m}}{K_{m}+L_{kk}^{i}\left(K_{c,kk}^{i}-K_{m}\right)}$$

(9) $${\langle \cos^{2}\theta \rangle }^{i}=\frac{\int \rho^{i}\left(\theta \right)\cos^{2}\theta \sin\theta d\theta }{\int \rho^{i}\left(\theta \right)\sin\theta d\theta }$$

(10) $$\gamma_{kk}^{i}=\left\{ \begin{array}{l} \left(2+1/p^{i}\right)\alpha_{k},\;\text{for}\;p^{i}\geq 1\\ \left(1+2p^{i}\right)\alpha_{k},\;\text{for}\;p^{i}<1 \end{array}\right.$$

(11) $$L_{33}^{i}=1-2L_{11}^{i}$$

(12) $$\alpha_{k}^{i}=R_{TB}^{i}K_{m}/a_{k}^{i}$$

(13) $$p^{i}=a_{3}^{i}/a_{1}^{i}$$

where *φ_i_* is the volume fraction, $a_{1}^{i}$ and $a_{3}^{i}$ are the particle radii, *p^i^* is the aspect ratio, $R_{TB}^{i}$ is the interfacial thermal resistance, *K_inc__.i_* is the thermal conductivity of inclusion of type *i*, *K_mat_* is the thermal conductivity of the matrix, and ${\langle \cos^{2}\theta \rangle }^{i}$ is a factor defining the orientation of inclusion of type *i*.

### 2.2. Effect of Nanometer-Sized Inclusions on Matrix and Inclusion Thermal Conductivities

The effect of multiple nanometer-sized inclusions oriented in any random direction on the thermal conductivities of the matrix and inclusions was calculated by extending the approach of Minnich and Chen [18]. According to their approach, the addition of the nanometer-sized particles modifies the thermal conductivities of the matrix and the inclusions. The modified thermal conductivities are calculated by first calculating the effective mean-free-path of the energy carriers (phonons or electrons) in the matrix or the inclusions using Matthiessen’s rule.

(14) $$\frac{1}{\Lambda_{x,eff}^{y}}=\frac{1}{\Lambda_{x,bulk}^{y}}+\frac{1}{\Lambda_{x,coll}^{y}}$$

where *x* can be *p* or *e* for phonons or electrons respectively and *y* can be *mat* for the matrix or *inc.i* for the *i*th inclusion.

The bulk mean-free-paths, $\Lambda_{x,bulk}^{y}$ for a material are known quantities. The unknown quantity in Equation (14) is the collision mean-free-path, $\Lambda_{coll}^{y}$, defined as the average distance traveled by the energy carriers between collisions. Once the effective mean-free-paths of the energy carriers have been calculated, the modified thermal conductivities of the matrix and inclusions can be calculated using,

(15) $$K_{x}^{y}=\frac{1}{3}C_{x}^{y}\nu_{x}^{y}\Lambda_{x,eff}^{y}$$

To calculate the collision mean-free-paths of the matrix, $\Lambda_{coll}^{mat}$, the densities of the inclusions in the matrix, *n_i_*, were calculated using Equation (16).

(16) $$n_{i}=\frac{N}{V}=\frac{\varphi_{i}}{V_{i}}$$

where *N* is the number of inclusions in a sample of volume *V* and $V_{i}$ is the volume of a single particle of inclusion type *i*.

To determine the collision mean-free-path of the matrix, it was noted that the number of inclusions of type *i* that an energy carrier (phonon or electron) encounters in a volume $A_{⊥1}L$ is $n_{i}A_{⊥1}L$ assuming $A_{⊥1}=\max\left(A_{⊥1},A_{⊥2},A_{⊥3},\ldots ,A_{⊥N}\right)$ where $A_{⊥\;i}$ is the collision cross-section area of the *i*th inclusion type. The matrix collision mean-free-path is therefore,

(17) $$\Lambda_{coll}^{mat}=\frac{L}{(n_{1}+n_{2}+\ldots +n_{N})A_{⊥1}L}$$

By replacing the expressions for *n_i_* in Equation (17), we get,

(18) $$\Lambda_{coll}^{mat}=\frac{1}{\left(\varphi_{1}+r_{V2}\varphi_{2}+\ldots +r_{VN}\varphi_{2}\right)\sigma_{⊥1}}$$

where $r_{Vi}=V_{1}/V_{i}$ and $\sigma_{⊥1}=A_{⊥1}/V_{1}$.

Equation (18) reduces to Ordonez-Miranda and coworkers’ formulation [19] when only a single inclusion was considered; that is when $\varphi_{i}=0\;\text{for}\;i=2,3,\ldots ,N$. It is also interesting to note that in Equation (18), the relative sizes of the inclusions, represented by $r_{Vi}$, determines their contribution to the collision mean-free-path for the matrix.

Applying Matthiessen’s rule, the effective mean-free-path of energy carriers in the matrix was estimated as,

(19) $$\Lambda_{x,eff}^{mat}=\frac{\Lambda_{x,bulk}^{mat}}{1+\Lambda_{x,bulk}^{mat}\left(\varphi_{1}+r_{V2}\varphi_{2}+\ldots +r_{VN}\varphi_{2}\right)\sigma_{⊥1}}$$

The modified thermal conductivity of the matrix material was calculated using Equation (15), which led to Equation (20).

(20) $$K_{x}^{mat}=\frac{K_{x,bulk}^{mat}}{1+\Lambda_{x,bulk}^{mat}\left(\varphi_{1}+r_{V2}\varphi_{2}+\ldots +r_{VN}\varphi_{2}\right)\sigma_{⊥1}}$$

The total matrix thermal conductivity is given by Equation (21).

(21) $$K_{mat}=K_{p}^{mat}+K_{e}^{mat}$$

For the calculation of the modified matrix thermal conductivity, the collision cross-sectional area of the inclusion is required. For the general case of a spheroidal inclusion oriented at any angle, Equation (22) was derived for the calculation of the collision cross-sectional area. The detailed derivation for Equation (22) is presented in Appendix A.

(22) $$A_{⊥1}=\pi b_{1}b_{2}$$

where,

(23) $$\begin{array}{l} b_{1}=a_{1}^{1}\\ b_{2}=\frac{a_{1}^{1}a_{3}^{1}}{\sqrt{a_{1}^{1}\sin^{2}\theta^{1}+a_{3}^{1}\cos^{2}\theta^{1}}} \end{array}$$

and $\theta^{1}$ is the average orientation angle of inclusion 1.

**Figure 2:**
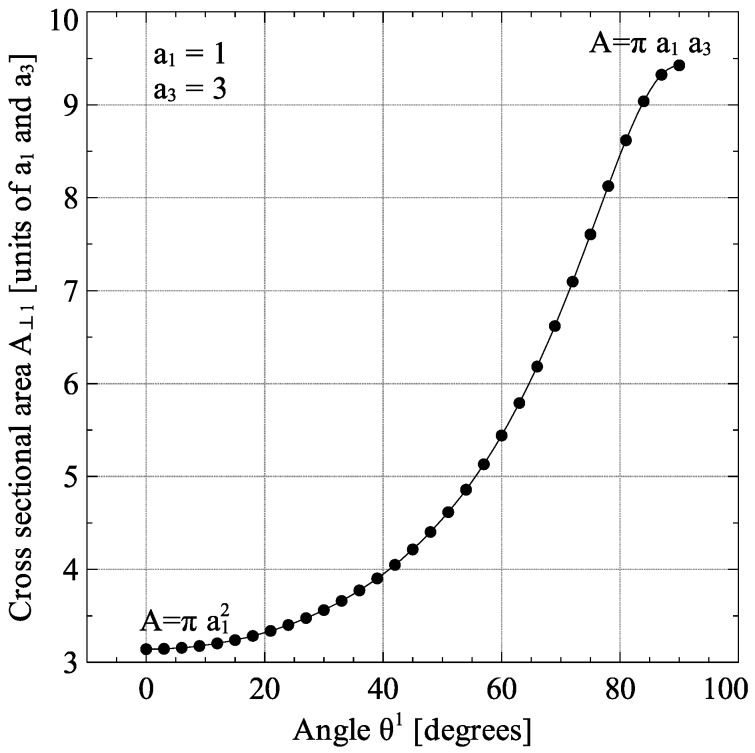
Variation of collision cross-sectional area $A_{⊥1}$ with angle *θ*^1^ for a spheroidal inclusion.

The dependence of the spheroidal cross section area $A_{⊥1}$ on $\theta^{1}$ is shown in Figure 2.

**Figure 3:**
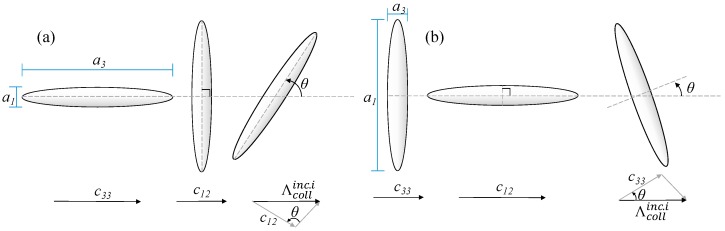
Calculating $\Lambda_{coll}^{inc.\;i}$ for: (**a**) prolate inclusion; and (**b**) oblate inclusion.

**Figure 4:**
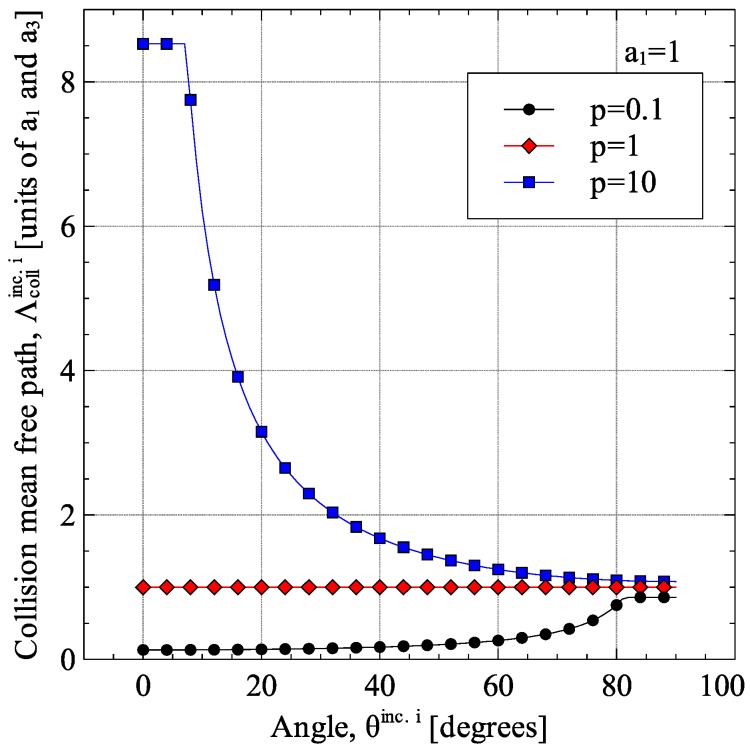
Variation of collision mean-free-path $\Lambda_{coll}^{int.i}$ with angle $\theta^{inc.i}$.

For the inclusions, the collision mean-free-path depends only on the size and orientation of the inclusion itself. For the special cases of inclusion aligned parallel ($\theta^{i}=0^{\circ }$) or perpendicular ($\theta^{i}=90^{\circ }$) to the direction of heat conduction, the collision mean-free-path for inclusion particles can be calculated using Equations (25) or (26), respectively [19]. For the general case of particles oriented at any angle between 0° and 90°, Equation (24) were derived to calculate the collision mean-free-path of inclusion particles. Figure 3 shows the reasoning behind Equation (24). For randomly oriented inclusions, the average orientation of the inclusions was estimated from ${\langle \cos^{2}\theta \rangle }^{i}=1/3$ as $\langle \theta^{i}\rangle =\cos^{-1}\left(1/\sqrt{3}\right)$. The variation of $\Lambda_{coll}^{inc.i}$ with angle $\theta^{i}$ is shown in Figure 4 for spheroids of aspect ratios 0.1, 1 and 10.

(24) $$\Lambda_{coll}^{inc.i}=\left\{ \begin{array}{l} \min\left(\frac{c_{12}^{inc.i}}{\sin\theta^{i}},c_{33}^{inc.i}\right),\;\text{for}\;p\geq 1\\ \min\left(\frac{c_{33}^{inc.i}}{\cos\theta^{i}},c_{12}^{inc.i}\right),\;\text{for}\;p<1 \end{array}\right.$$

(25) $$c_{12}^{inc.i}=\left\{ \begin{array}{l} \frac{8a_{1}^{i}}{3\pi \varepsilon }\frac{\left(1+\varepsilon^{2}\right)K_{ellip}\left(-\varepsilon^{2}\right)-\left(1-\varepsilon^{2}\right)E_{ellip}\left(-\varepsilon^{2}\right)}{\varepsilon \sqrt{1+\varepsilon^{2}}+\text{arcsinh}\left(\varepsilon \right)},\;\text{for}\;p^{i}<1\\ \frac{8a_{1}^{i}}{3\pi \left(i\varepsilon \right)}\frac{\left(1-\varepsilon^{2}\right)K_{ellip}\left(\varepsilon^{2}\right)-\left(1+\varepsilon^{2}\right)E_{ellip}\left(\varepsilon^{2}\right)}{\left(i\varepsilon \right)\sqrt{1-\varepsilon^{2}}+\text{arcsinh}\left(i\varepsilon \right)},\;\text{for}\;p^{i}\geq 1 \end{array}\right.$$

(26) $$c_{33}^{inc.i}=\left\{ \begin{array}{l} \frac{4a_{3}^{i}}{3\varepsilon }\frac{\sqrt{{\left(1+\varepsilon^{2}\right)}^{3}}-1}{\varepsilon \sqrt{1+\varepsilon^{2}}+\text{arcsinh}\left(\varepsilon \right)},\;\text{for}\;p^{i}<1\\ \frac{4a_{3}^{i}}{3\left(i\varepsilon \right)}\frac{\sqrt{{\left(1-\varepsilon^{2}\right)}^{3}}-1}{\left(i\varepsilon \right)\sqrt{1-\varepsilon^{2}}+\text{arcsinh}\left(i\varepsilon \right)},\;\text{for}\;p^{i}\geq 1 \end{array}\right.$$

where ε is the eccentricity given by,

(27) $$\varepsilon =\left\{ \begin{array}{l} \sqrt{1-p^{i^{2}}},\;\text{for}\;p^{i}<1\\ \sqrt{p^{i^{2}}-1},\;\text{for}\;p^{i}\geq 1 \end{array}\right.$$

and $K_{ellip}\left(\;\right)$ and $E_{ellip}\left(\;\right)$ are elliptic integrals of first and second kind.

The effective mean-free-path of the energy carriers in the inclusion particles was calculated by applying the Matthiessen’s rule.

(28) $$\Lambda_{x,eff}^{inc.i}=\frac{\Lambda_{x,bulk}^{inc.i}}{1+\Lambda_{x,bulk}^{inc.i}/\Lambda_{coll}^{inc.i}}$$

By replacing the effective mean-free-path in Equations (15) for thermal conductivity, the reduced electron or phonon thermal conductivities of the inclusions were derived using Equation (29).

(29) $$K_{x}^{inc.i}=\frac{K_{x,bulk}^{inc.i}}{1+\Lambda_{x,bulk}^{inc.i}/\Lambda_{coll}^{inc.i}}$$

The total thermal conductivity for inclusion *i* is the sum of the phonon and electron thermal conductivities given by Equation (30).

(30) $$K_{inc.i}=K_{p}^{inc.i}+K_{e}^{inc.i}$$

### 2.3. Two-Scale Approach for Non-Uniformly Distributed Inclusions

A major drawback of the effective medium theory approach is its inability to handle non-uniform dispersion of inclusions. In the current work, a two-scale approach was proposed that combines the EMT approach at the lower scale with computational homogenization approach at the upper scale [30,31] to introduce the effect of non-uniform dispersion of inclusions in the matrix on the effective thermal conductivity of the composite.

**Figure 5:**
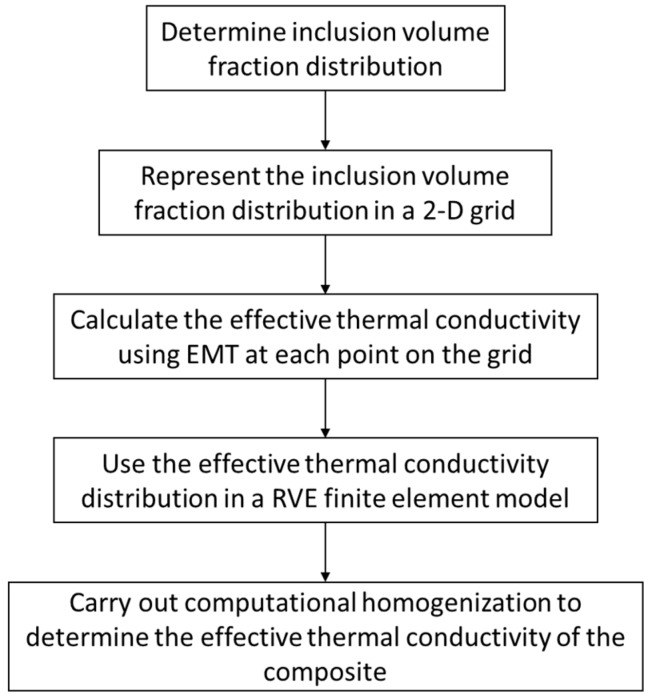
Two-scale methodology for effective thermal conductivity estimation.

The application of the method requires a quantitative knowledge of inclusion distribution in the matrix. This could be a statistical parameter like standard deviation of inclusion volume fraction inside the matrix that can be used to develop a representative volume element (RVE) for computational homogenization. The distribution could also be experimentally determined using material characterization techniques. The key point in the application of the two-scale methodology is the requirement of scale separation between the size scale of inclusions in the composites and the scale at which the homogenized properties are being estimated. This means that the distribution needs to be measured at a length scale larger than the inclusion size. As an example, if the inclusions are nanometer-sized, the distribution needs to be determined at the micrometer length scale. The two-scale methodology is presented in Figure 5.

**Figure 6:**
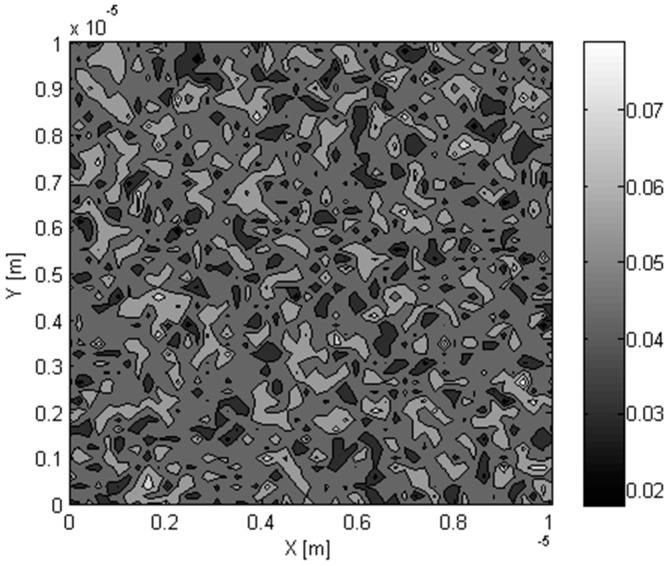
Distribution of Si nanoparticle volume fraction in Ge matrix.

**Figure 7:**
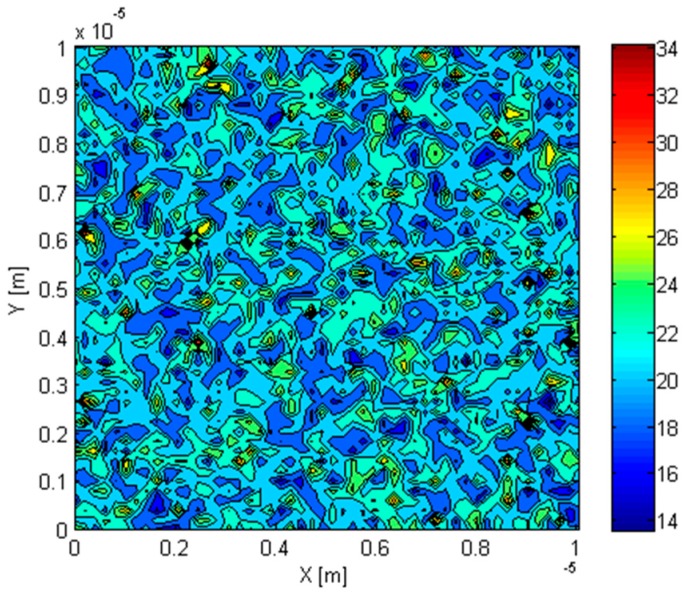
Distribution of the effective thermal conductivity in the RVE.

In the first two steps in the methodology, the variation of inclusion volume fraction at different points in the composite is determined and represented in the form of a grid of points for each inclusion. As a result, the distribution of inclusions in the composite can be shown as in Figure 6, which shows the distribution of a nanometer sized Si inclusion in Ge matrix. Because of the scale separation requirement, each point in the grid represents the composite material having a certain volume fraction of inclusions. In the example considered in the figure, the average inclusion volume fraction in the Ge–Si RVE was 5% and the standard deviation of the volume fraction in the microscale domain was 1%. The distribution of the effective thermal conductivity in the RVE determined using the generalized effective medium theory is shown in Figure 7.

The final step in the methodology is to apply computational homogenization to estimate the overall effective thermal conductivity of the composite. An important aspect of the accuracy of computational homogenization results is the correct determination of the RVE size required for homogenization. In the current work, the validity of the RVE size used was determined using the methodology presented by Gitman and coworkers [32]. Gitman and coworkers suggested the calculation of a variation coefficient, known as the *chi-square criterion* (*χ*^2^) using Equation (31). If the *chi-square criterion* is below a threshold value, the homogenization results are considered to be independent of RVE size. A threshold of 0.1 was selected in the present study as suggested by Gitman and coworkers

(31) $$\chi^{2}=\frac{\sum_{i=1}^{n}{\left(a_{i}-\langle a\rangle \right)}^{2}}{\langle a\rangle }$$

where *a_i_* is the homogenized property under consideration for RVE realization *i*, *n* is the total number of random RVE realizations of the same size and 〈a〉 is the mean value of the property under consideration.

**Figure 8:**
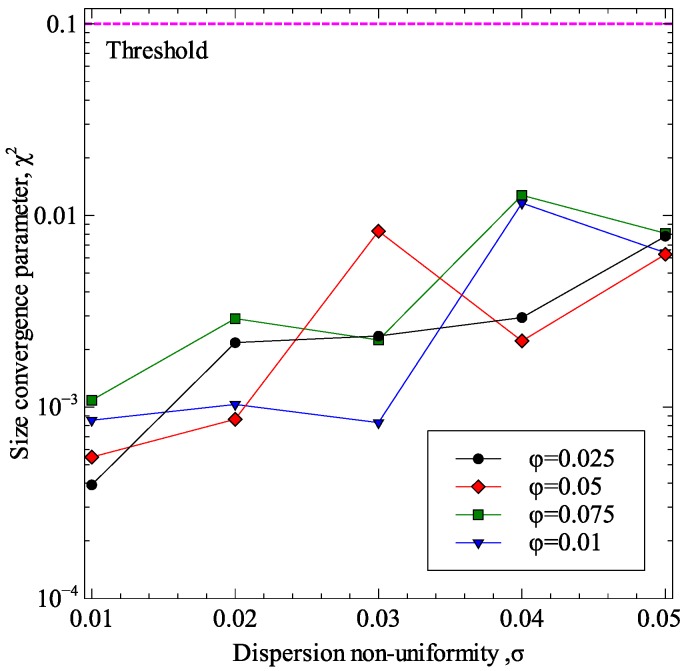
Verification of RVE size for computational homogenization.

For the computational homogenization problem of the current work, the parameter *χ*^2^ was determined for multiple average inclusion volume fractions and multiple dispersion non-uniformity values (modeled using standard deviation of inclusion volume fraction). The results of this study are presented in Figure 8. From the results, it was concluded that the use of RVEs of 10 μm edge length will result in homogenized thermal conductivity values which are independent of the RVE size. Therefore, this RVE size was used.

**Figure 9:**
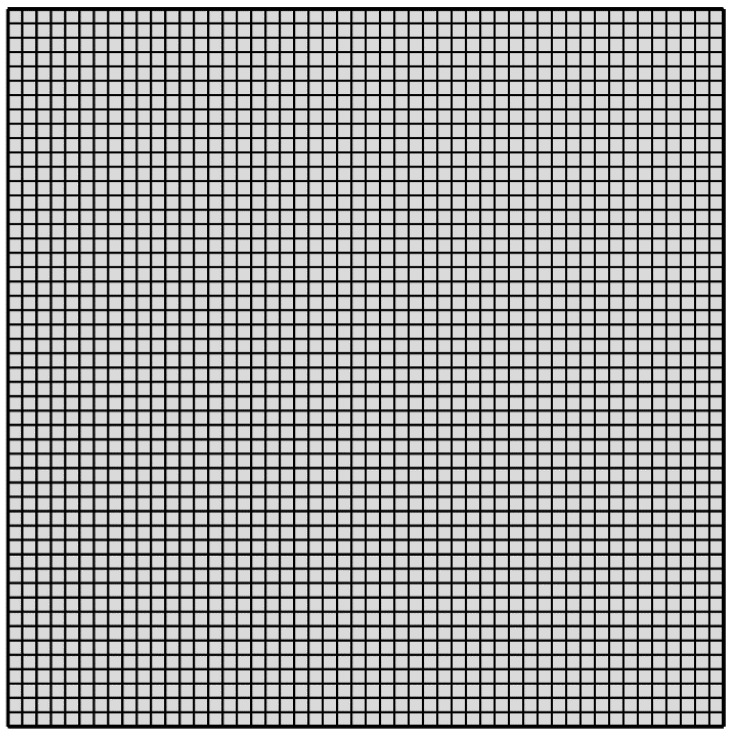
Finite element mesh for computational homogenization.

Computational homogenization was carried using COMSOL/MATLAB. A mesh convergence study was conducted to determine the number of elements required for results to be independent of the element size. It was found that increasing the number of elements beyond 50 × 50 did not result in any significant change in the results. The final mesh having 50 × 50 elements used in the current work to carry out computational homogenization is shown in Figure 9.

The effect of distribution non-uniformity was incorporated into the mesh using an interpolation function which applied the thermal conductivity distribution, as shown in Figure 7, to the integration points in the finite element mesh. It is important to note here that due to the condition of scale separation, the inclusion particles were not explicitly modeled in the geometry of the finite element domain. A temperature gradient was applied along the x- and y-directions of the RVE and Equation (32) was used to determine the effective thermal conductivity of the composite.

(32) $$K_{eff}=\frac{{\langle q\rangle }_{11}{\langle \nabla T\rangle }_{11}+{\langle q\rangle }_{22}{\langle \nabla T\rangle }_{22}+{\langle q\rangle }_{33}{\langle \nabla T\rangle }_{33}}{{{\langle \nabla T\rangle }_{11}}^{2}+{{\langle \nabla T\rangle }_{22}}^{2}+{{\langle \nabla T\rangle }_{33}}^{2}}$$

where *q* is the heat flux, ∇T is the temperature gradient, and $\langle •\rangle =\frac{1}{\left|V\right|}\int_{V}•dV$ and *V* is the domain volume.

For the example of 5% Ge–Si composite considered above, the overall effective thermal conductivity of the composite was determined to be 21.41 W/m·K. For the case when inclusion distribution was uniform, the effective thermal conductivity was determined to be 21.06 W/m·K.

## 3. Applications of the Generalized Effective Medium Theory

**Figure 10:**
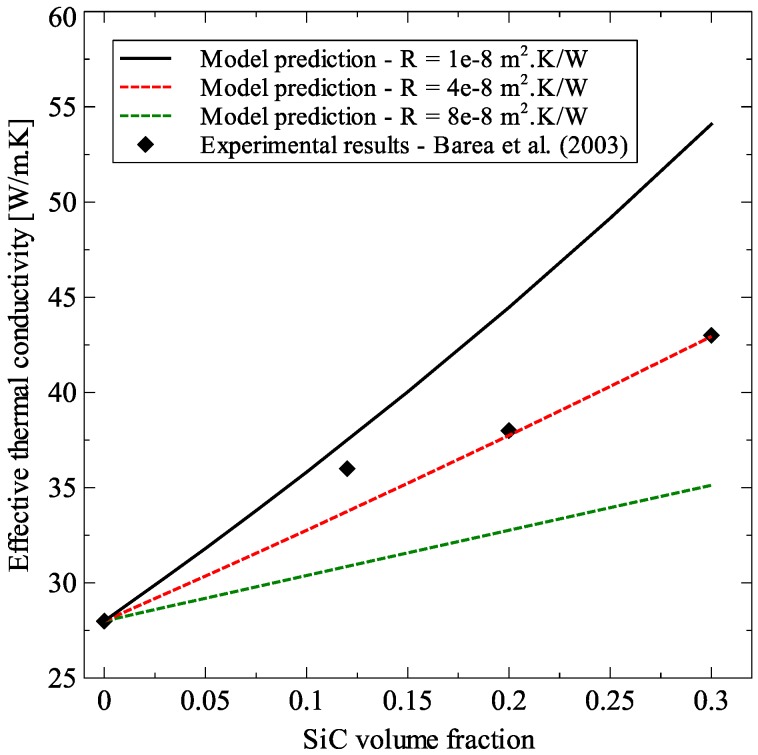
Comparison of model predictions with experimental results for Al_2_O_3_–SiC composite [33].

**Figure 11:**
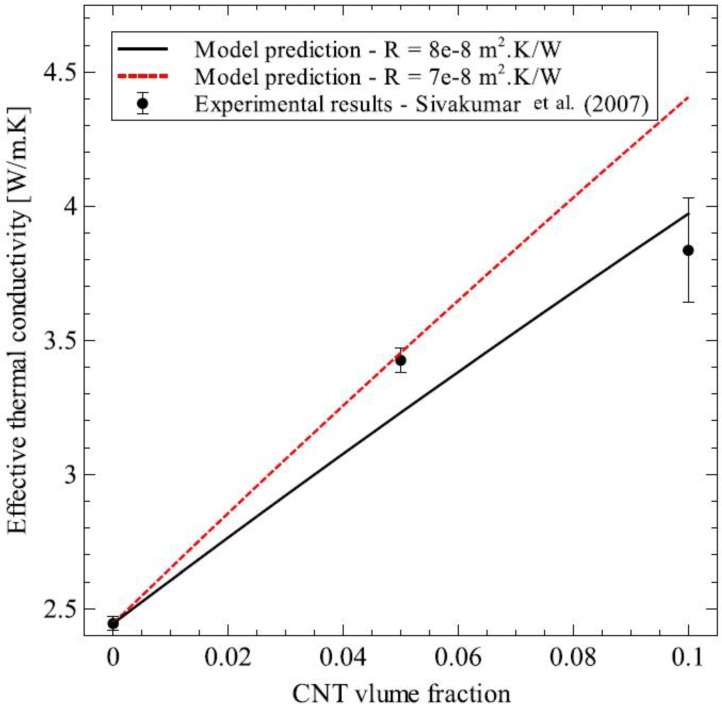
Comparison of model predictions with experimental results for SiO_2_–CNT composite [34].

In this section, the generalized effective medium theory formulated in the previous section was applied to different particulate composites to show its various capabilities. Before the studies were carried out, the model was validated against two experimental datasets for Al_2_O_3_–SiC platelet composite [33] and SiO_2_–CNT composite [34]. The results of these validations are presented in Figure 10 and Figure 11 for Al_2_O_3_–SiC composite and SiO_2_–CNT composite, respectively. For both validations, the matrix thermal conductivity was set as the experimentally determined value and the inclusion dimensions were taken from the original experimental data. The model predictions were calculated by varying the thermal interface resistance in the range of 1 × 10^−8^ to 8 × 10^−8^ m^2^·K/W. This was done to take into account the variation in thermal interface resistance that can occur due to change in process parameters, the source of raw materials and can even vary greatly from sample to sample [35]. Model predictions for both composites showed good agreement with experimental measurements.

### 3.1. Effect of Nanometer-Sized Inclusions

The generalized EMT formulated in the current work was applied to three particulate nanocomposites, Ge–Si composites, alumina–CNT composites and aluminum–CNT composites, to study the effect of nanometer-sized inclusions on the effective thermal conductivity of the composites.

**Figure 12:**
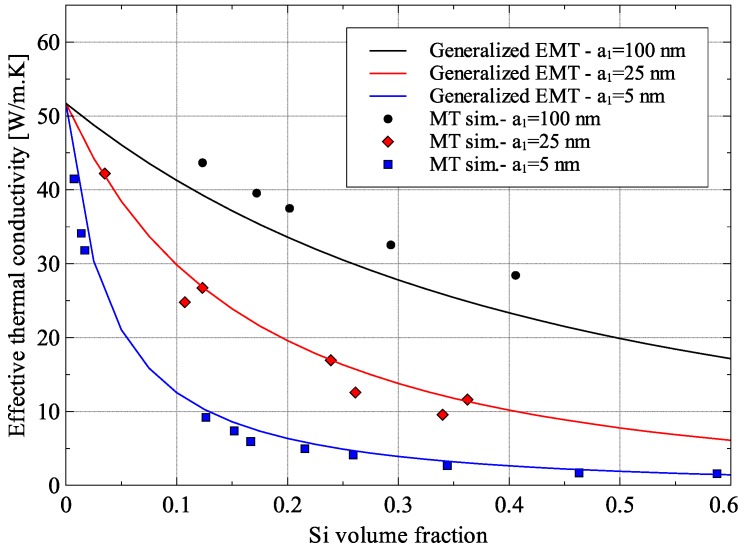
Predicted effective thermal conductivity of Ge–Si nanocomposite compared to Monte Carlo (MT) simulations [18] (*R_TB_* = 6.80 × 10^−9^ m^2^·K/W).

The Ge–Si composite was studied for a case reported in the literature for spherical Si inclusions in Ge matrix. The thermal interface resistance, *R*, was calculated using the diffuse mismatch model given by Equation (33) and Si and Ge material properties were taken from [14] and are given in Table 1. Figure 12 shows a comparison of the effective thermal conductivities of Ge–Si composite predicted by the generalized EMT formulation with those predicted by Monte Carlo simulations [14].

(33) $$R_{TB}=\frac{4\left(C_{m}\nu_{m}+C_{p}\nu_{p}\right)}{C_{m}\nu_{m}C_{p}\nu_{p}}$$

The results showed that the effective thermal conductivity of Ge–Si nanocomposites is significantly lower than the thermal conductivities of Si and Ge. The effective thermal conductivity falls to less than 5 W/m·K for Si inclusions with a diameter equal to 10 nm and a volume fraction of 20%. This drastic reduction occurs because both Silicon and Germanium have mean-free-paths much greater than the size of the Si inclusion.

The root-mean-squared error (RMSE) in model predictions for the nanocomposite with Si particles of radii 5 nm, 25 nm and 100 nm were 1.68 W/m·K, 2.18 W/m·K, and 4.45 W/m·K, respectively. These translated to normalized errors of 12.72%, 10.22%, and 12.25%, respectively. The RMSE for the case with inclusion size of 100 nm was higher than the other cases because the thermal conductivity of the composite was higher for this case. On the other hand, the normalized root mean squared error all three cases were almost the same. That being said, the difference in the results of the Monte Carlo simulation and the generalized EMT can be attributed to a number of reasons including the difference in the details included in the models and model domain size. Monte Carlo simulations involve the detailed simulation of the transport of a large number of energy carriers through the material whereas the generalized EMT only models the average effect of transport of energy carriers. But because of their complexity, Monte Carlo simulations are extremely computationally intensive and therefore are usually solved over a unit cell containing a single inclusion particle.

**Figure 13:**
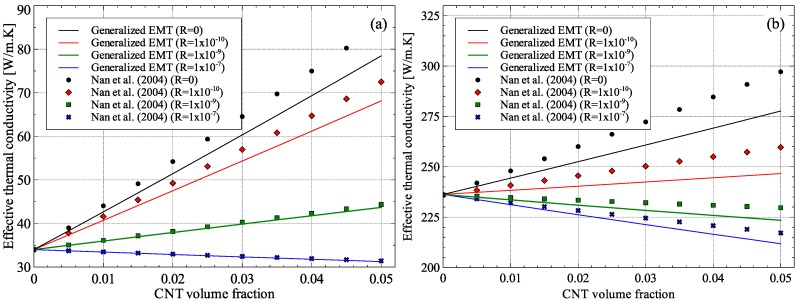
Effective thermal conductivity of: (**a**) Alumina–MWCNT; and (**b**) Aluminum–MWCNT nanocomposites.

The generalized EMT was also applied to the case to alumina and aluminum matrix composites with randomly oriented multi-walled carbon nanotubes (MWCNT). A comparison between the generalized EMT and Nan and coworkers’ model [36] for the two cases is shown in Figure 13. Nan and coworkers’ model can take into account the effect of size and orientation of CNTs on the effective thermal conductivity of the composite but ignores the effect of CNT size on the thermal conductivity of matrix and CNT inclusion itself. The properties of alumina, aluminum, and MWCNTs used in the current work are shown in Table 2.

For comparison between the two models, the value of the thermal interface resistance, *R*, was varied from 0 to 1 × 10^−7^. For high values of thermal interface resistance, a very small difference was observed between the generalized EMT and Nan and coworkers’ model for alumina–MWCNT composite. In this case, the effect of interfacial resistance dominated the effect of inclusion size and therefore, the difference is more prominent at lower values of *R*. On the other hand, the aluminum–MWCNT composites showed dependence on CNT size for all interfacial resistance values. A significant result of the comparison is that the theoretical maximum effective thermal conductivity possible when R=0 is reduced from 85 W/m·K to 78 W/m·K for alumina matrix composite and from 298 W/m·K to 278 W/m·K for aluminum matrix composite when the CNT volume fraction is 5%.

**Figure 14:**
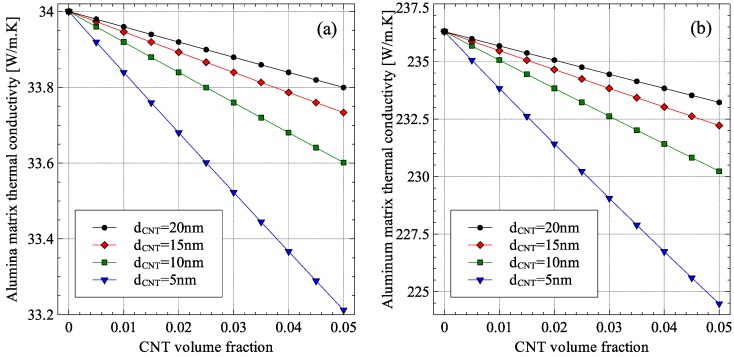
Modified (**a**) alumina matrix and (**b**) aluminum matrix thermal conductivities in MWCNT nanocomposites with randomly oriented CNTs.

To investigate the reason for the difference in the effect of nanometer inclusion sizes on the effective thermal conductivity of alumina–CNT and aluminum–CNT composites, the variation of the reduced thermal conductivities of alumina and aluminum matrices with CNT diameter was analyzed. The results are shown in Figure 14. As shown in the figure, the CNT diameter had a minimal effect on the alumina matrix thermal conductivity, which dropped by around 1 W/m·K when the CNT diameter was reduced from 20 nm to 5 nm. For the same change in CNT diameter, the thermal conductivity of the aluminum dropped from 236 W/m·K to 224.5 W/m·K. This large difference between aluminum and alumina in the sensitivity to CNT size can be attributed to the difference in the mean-free-paths of energy carriers for aluminum and alumina. For aluminum, the dominant energy carriers are the electrons whose mean-free-path is 14.1 nm compared to the 4.6 nm phonon mean-free-path for alumina. Since the CNT diameters were larger than the phonon mean-free-path for alumina, the addition of CNTs had little effect on the thermal conductivity of alumina.

### 3.2. Effect of Inclusion Aspect Ratio

**Figure 15:**
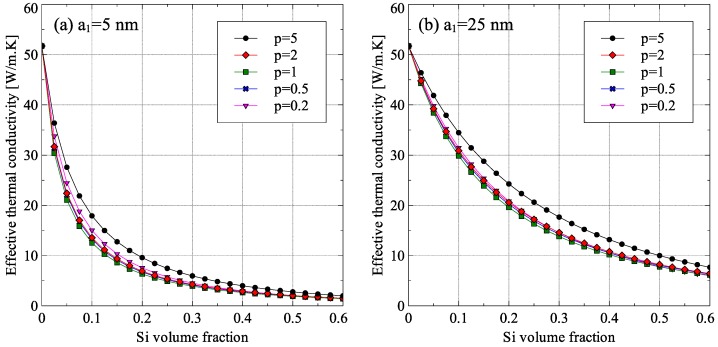
Dependence of Ge–Si effective thermal conductivity on inclusion aspect ratio *p* for inclusion size $a_{1}$ equal to: (**a**) 5 nm; and (**b**) 25 nm (*R_TB_* = 6.80 × 10^−9^ m^2^·K/W).

The generalized EMT was also used to study the effect of inclusion aspect ratio on the effective thermal conductivity of randomly oriented spheroidal inclusions $\left(\langle \cos^{2}\theta \rangle =1/3\right)$ using the example of Ge–Si nanocomposite. The results are shown in Figure 15. To compare the inclusions of various aspect ratios, it was assumed that the volume of a single inclusion is same for all cases. The results in Figure 15 show that for inclusions having same volume, the best thermal conductivity reduction was achieved for spherical inclusions. The reason for this was that spherical inclusions had the largest cross-sectional area to obstruct the path of the energy carriers in the matrix (1963.5 nm^2^ for the inclusion of radius 25 nm as compared to 1119.2 nm^2^ and 1392.5 nm^2^ for inclusions of aspect ratios 0.2 and 5, respectively). This resulted in greater reduction in thermal conductivity for spherical inclusions.

### 3.3. Effect of Inclusion Orientation

**Figure 16:**
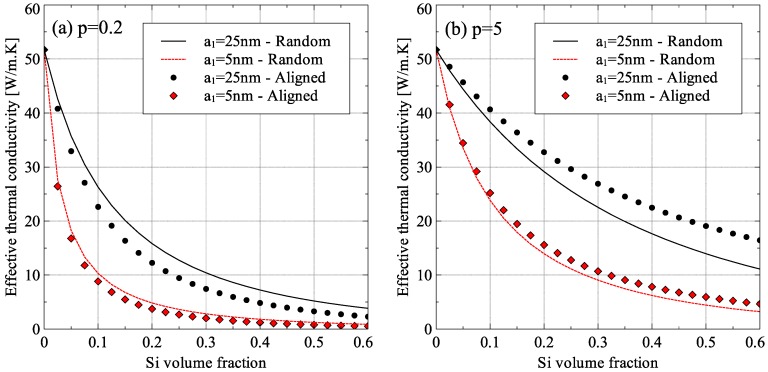
Dependence of Ge–Si effective thermal conductivity on inclusion alignment for inclusion aspect ratio *p* equal to: (**a**) 0.2; and (**b**) 5 (*R_TB_* = 6.80 × 10^−9^ m^2^·K/W).

The effective thermal conductivity of aligned spheroidal nanometer-sized inclusions was estimated by Ordonez-Miranda and coworkers [19]. The generalized EMT presented in the current work extends their approach to composites with randomly oriented spheroidal inclusions. A comparison of the axial direction effective thermal conductivity of Ge–Si nanocomposites with aligned and randomly oriented inclusion is presented in Figure 16, for two different aspect ratios of inclusions. For oblate inclusions (*p* < 1), the alignment of inclusions in a specific direction resulted in reduced effective thermal conductivity, which is the main purpose of the development of Ge–Si nanocomposites. On the other hand, for prolate inclusions (*p* > 1), the effect was reversed and randomly oriented prolate inclusions were more effective in reducing the thermal conductivity of Ge–Si composites.

The difference in the behavior of oblate and prolate shaped inclusions was because of two reasons. First, the cross-sectional area of the inclusion obstructing the motion of energy carriers in the matrix was reduced due to random orientation of particles in case of oblate inclusions while it increased for prolate inclusions. This resulted in the matrix thermal conductivity to increase due to random orientation of oblate inclusion and reduce due to the random orientation of prolate inclusions. Second, the collision mean-free-path of the energy carriers inside the inclusion increased due to random orientation of oblate inclusions while it reduced due to the random orientation of prolate inclusions. This resulted in an increase in the inclusion thermal conductivity for oblate inclusions and a decrease in the inclusion thermal conductivity for prolate inclusions due to random orientation.

### 3.4. Effect of Non-Uniform Dispersion of Inclusions

**Figure 17:**
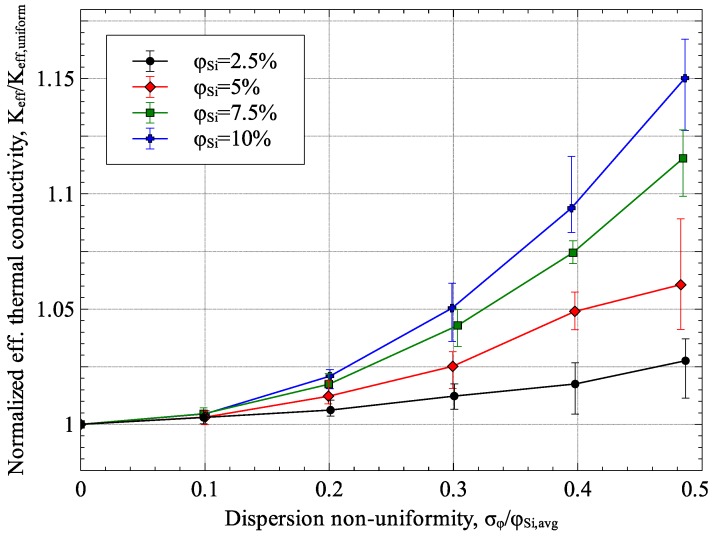
Effect of non-uniform dispersion on Ge–Si effective thermal conductivity (*R_TB_* = 6.80 × 10^−9^ m^2^·K/W).

The two-scale approach presented in the current was used to study the effect of non-uniform dispersion on the effective thermal conductivity of Ge–Si nanocomposites. Without loss of generality, Ge–Si nanocomposites with 2.5%, 5%, 7.5% and 10% Si inclusions with a diameter equal to 10 nm were considered in the study. The results, presented in Figure 17, show the effective thermal conductivity of the Ge–Si nanocomposite normalized with effective thermal conductivity of Ge–Si nanocomposite with uniformly dispersed inclusions plotted against the non-uniformity in inclusion distribution (shown in the figure as the ratio of standard deviation, *σ_φ_*, and average inclusion volume fraction, *φ*_Si,avg_). To analyze the variation of results due to randomness, each case was repeated five times by randomly generating an inclusion distribution in the RVE. As can be seen from the figure, the effectiveness of Si inclusions in reducing the effective thermal conductivity of the composite is adversely affected by non-uniformity in inclusion dispersion.

## 4. Sensitivity Analysis of the Generalized Effective Medium Theory

To identify the relative importance of the material properties and other parameters used in the generalized effective medium theory, a sensitivity analysis was carried. The model parameters considered in the sensitivity analysis were the matrix and inclusion thermal conductivities, mean-free-path of the energy carriers in the matrix and inclusion, the inclusion volume fraction and the thermal interface resistance. Using the case of Ge-20% Si nanocomposite as the nominal case, normalized sensitivity coefficients (NSCs) were calculated for each parameter. Normalized sensitivity coefficients, calculated using Equation (34) express the order of magnitude change in the analyzed function that will result from one order of magnitude change in the concerned parameter [37,38]. Using normalized sensitivity coefficients, one-to-one comparison between the model parameters can be carried out and parameters to which the model is more sensitive to can be determined.

(34) $$NSC_{i}=\frac{\Delta Y}{\bar{Y}}\frac{{\bar{X}}_{i}}{\Delta X_{i}}$$

where $\bar{Y}$ is nominal value of the function at nominal model parameters ${\bar{X}}_{i}$ and ΔY is the change in the function value to due to a change of $\Delta X_{i}$ in the model parameter $X_{i}$.

**Figure 18:**
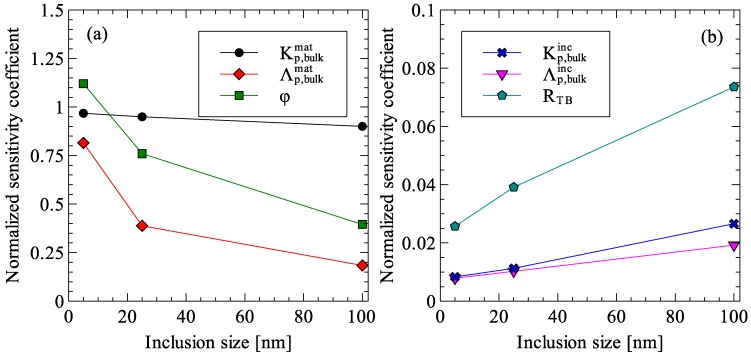
Variation of normalized sensitivity coefficients for: (**a**) bulk matrix thermal conductivity, matrix phonon mean-free-path and inclusion volume fraction; and (**b**) bulk inclusion thermal conductivity, inclusion phonon mean-free-path and thermal interface resistance.

For the case of Ge–Si nanocomposite, the results of the sensitivity analysis are presented in Table 3 for three inclusion sizes and the variation of the normalized sensitivity coefficients with inclusion size is presented in Figure 18. The results of the study showed that the NSCs of three model parameters, bulk matrix thermal conductivity, matrix phonon mean-free-path and inclusion volume fraction, were at least an order of magnitude higher than the NSCs of the remaining three parameters. This implies that the effective thermal conductivity of the Ge–Si nanocomposite is an order of magnitude more sensitive to changes in these three parameters. An analysis of Figure 18 also revealed several important points regarding the sensitivity of the generalized effective medium theory to the model parameters. First, the NSC of the bulk matrix thermal conductivity remained almost constant for inclusion radii from 5 nm to 100 nm. This means the sensitivity of the effective thermal conductivity of the composite is independent of the inclusion size. Second, with an increasing inclusion size, the NSCs of matrix phonon mean-free-path and the inclusion volume fraction decreased while the NSC of the thermal interface resistance showed a significant increase. Both inclusion volume fraction and matrix phonon mean-free-path affect the reduction in the matrix thermal conductivity due to nanometer sized inclusion. This suggests that with an increase in the inclusion size, the effect of interfacial thermal resistance starts to dominate the effect of reduced matrix thermal conductivity.

## 5. Conclusions

In this paper, we presented a generalized effective medium theory for the estimation of the effective thermal conductivity of particulate nanocomposites. The formulated EMT has the capability of incorporating the effects of size, shape, orientation and dispersion non-uniformity of multiple inclusions on the estimated thermal conductivity of particulate composites. Several applications of the formulated EMT were also presented.

For the Ge–Si nanocomposite, it was found that spherical Si inclusions result in better effective thermal conductivity reduction in the nanocomposite. It was also found that aligned oblate inclusions result be better thermal conductivity reduction than randomly oriented oblate inclusions. The effect is reversed for the case of prolate inclusions for which randomly oriented prolate inclusions show better thermal conductivity reduction. Finally, the effective thermal conductivity was found to be strongly dependent on the dispersion uniformity of the inclusion particles for Ge–Si nanocomposites. The effectiveness of nanometer-sized Si particles in reducing the thermal conductivity of Ge matrix reduced with increasing non-uniformity in Si dispersion.

For alumina–MWCNT and aluminum–MWCNT nanocomposites, the effect of high scattering of energy carriers on the effective thermal conductivity increased with reducing interface thermal resistance. Between alumina and aluminum, aluminum showed a greater sensitivity to CNT size due to its relativity large electron mean-free-path.

A sensitivity analysis carried out to determine the relative effective of the model parameters on the effective thermal conductivity of Ge–Si nanocomposites showed that the model was an order of magnitude more sensitive to changes in the matrix thermal conductivity, matrix phonon mean-free-path and inclusion volume fraction than changes in inclusion thermal conductivity, inclusion phonon mean-free-path and thermal interface resistance. The analysis also showed that as inclusion size increases, the model becomes increasingly sensitive to variation in thermal interface resistance.

## Figures and Tables

**Table 1:** Properties used for Ge–Si nanocomposite.

Material	Bulk Thermal Conductivity (W/m·K)	Bulk Phonon Mean-Free-Path (nm)	Phonon Group Velocity (m/s)	Volumetric Specific Heat Capacity (J/m^3^·K)
Silicon	150	268	1804	0.93 × 10^6^
Germanium	51.7	171	1042	0.87 × 10^6^

**Table 2:** Properties for alumina, aluminum, and MWCNTs.

Material	Bulk Phonon Thermal Conductivity (W/m·K)	Bulk Electron Thermal Conductivity (W/m·K)	Bulk Phonon Mean-Free-Path (nm)	Bulk Electron Mean-Free-Path (nm)	Diameter (nm)	Length (μm)
Alumina	34	-	4.6	-	-	-
Aluminum	11.3	225	2.3	14.1	-	-
MWCNT	3000	-	2.2	-	10	2

**Table 3:** Normalized sensitivity analysis results for generalized EMT.

**Parameter**	**a_1_ = 5 nm**			$\bar{Y}$ **= 6.3443 W/m·K**				
	$\bar{X}$	**X+**	**X−**	**Y+**	**Y−**	**ΔX**	**ΔY**	**NSC**
$K_{p,bulk}^{mat}$	51.70	56.87	46.53	6.98	5.75	10.34	1.23	0.97
$K_{p,bulk}^{inc}$	150.00	165.00	135.00	6.37	6.36	30.00	0.01	8.35 × 10^−3^
$\Lambda_{p,bulk}^{mat}$	1.71 × 10^−7^	1.88 × 10^−7^	1.54 × 10^−7^	5.89	6.92	3.42 × 10^−8^	1.04	0.82
$\Lambda_{p,bulk}^{inc}$	2.68 × 10^−7^	2.95 × 10^−7^	2.41 × 10^−7^	6.36	6.37	5.36 × 10^−8^	0.01	7.88 × 10^−3^
R_TB_	6.80 × 10^−9^	7.48 × 10^−9^	6.12 × 10^−9^	6.33	6.36	1.36 × 10^−9^	0.03	25.69 × 10^−3^
*φ*	0.20	0.22	0.18	5.70	7.12	0.04	1.42	1.12
**Parameter**	**a_1_ = 25 nm**			$\bar{Y}$ **= 19.58 W/m·K**				
	$\bar{X}$	**X+**	**X−**	**Y+**	**Y−**	**ΔX**	**ΔY**	**NSC**
$K_{p,bulk}^{mat}$	51.70	56.87	46.53	21.44	17.72	10.34	3.72	0.95
$K_{p,bulk}^{inc}$	150.00	165.00	135.00	19.60	19.56	30.00	0.04	11.26 × 10^−2^
$\Lambda_{p,bulk}^{mat}$	1.71 × 10^−7^	1.88 × 10^−7^	1.54 × 10^−7^	19.04	20.57	3.42 × 10^−8^	1.52	0.39
$\Lambda_{p,bulk}^{inc}$	2.68 × 10^−7^	2.95 × 10^−7^	2.41 × 10^−7^	19.56	19.60	5.36 × 10^−8^	0.04	10.27 × 10^−2^
R_TB_	6.80 × 10^−9^	7.48 × 10^−9^	6.12 × 10^−9^	19.51	19.66	1.36 × 10^−9^	0.15	39.15 × 10^−2^
*φ*	0.20	0.22	0.18	18.26	21.23	0.04	2.98	0.76
**Parameter**	**a_1_ = 100 nm**			$\bar{Y}$ **= 33.5943 W/m·K**				
	$\bar{X}$	**X+**	**X−**	**Y+**	**Y−**	**ΔX**	**ΔY**	**NSC**
$K_{p,bulk}^{mat}$	51.70	56.87	46.53	36.62	30.57	10.34	6.05	0.90
$K_{p,bulk}^{inc}$	150.00	165.00	135.00	33.68	33.50	30.00	0.18	26.60 × 10^−2^
$\Lambda_{p,bulk}^{mat}$	1.71 × 10^−7^	1.88 × 10^−7^	1.54 × 10^−7^	32.99	34.22	3.42 × 10^−8^	1.24	0.18
$\Lambda_{p,bulk}^{inc}$	2.68 × 10^−7^	2.95 × 10^−7^	2.41 × ^−7^	33.53	33.66	5.36 × 10^−8^	0.13	19.26 × 10^−2^
R_TB_	6.80 × 10^−9^	7.48 × 10^−9^	6.12 × 10^−9^	33.36	33.86	1.36 × 10^−9^	0.49	73.60 × 10^−2^
*φ*	0.2	0.22	0.18	32.30	34.96	0.04	2.65	0.40

## Appendix A. Calculation of the Collision Cross-Section Area

A general spheroidal inclusion particle placed at an angle θ in the direction of heat flow projects an area $A_{⊥}$ perpendicular to the direction of the heat flow. To calculate this cross-sectional area, we start with the general equation of an ellipsoid,

(A1) $${\left(x-c\right)}^{T}R^{T}AR\left(x-c\right)=1$$

where $x={\left[x,y,z\right]}^{T}$, **c** is the vector defining the center of the ellipsoid, **R** is the rotation matrix and **A** is 3 × 3 matrix with diagonal terms $\left(1/a_{1}^{2},1/a_{2}^{2},1/a_{3}^{2}\right)$. Assume a spheroid ($a_{1}=a_{2}$) centered at the origin ($c={\left[0,0,0\right]}^{T}$) and rotated about the y-axis by an angle θ (a rotation about the x-axis will provide the same result). To calculate the area of the ellipse projected on the global 12 plane, we need to calculate its major and minor axes of the projection. This can be done by replacing z=0 and determining the derivative dy/dx which is,

(A2) $$\frac{dy}{dx}=-\frac{x}{y}a_{1}^{2}\left(\frac{\cos^{2}\theta }{a_{1}^{2}}+\frac{\sin^{2}\theta }{a_{3}^{2}}\right)$$

x-intercepts can be determined by setting the numerator equal to zero which leads to the points $\left(0,\pm b_{1}\right)$. Similarly, y-intercepts are determined by setting the denominator equal to zero, which leads to the points $\left(\pm b_{2},0\right)$ where,

(A3) $$\begin{array}{l} b_{1}=a_{1}\\ b_{2}=\frac{a_{1}a_{3}}{\sqrt{a_{1}\sin^{2}\theta +a_{3}\cos^{2}\theta }} \end{array}$$

The area of the projected ellipse is therefore,

(A4) $$A_{⊥}=\pi b_{1}b_{2}$$
